# Dermoscopic Features of a Solitary Glomeruloid Hemangioma: A Rare Nonsyndromic Presentation

**DOI:** 10.7759/cureus.115994

**Published:** 2026-09-09

**Authors:** Sofia Elhaitamy, Sara Elloudi, Salma Kozmane, Layla Tahiri Elousrouti, Fatima Zahra Mernissi

**Affiliations:** 1 Department of Dermatology, Faculty of Medicine, Pharmacy, and Dental Medicine, Hassan II University Hospital, Sidi Mohamed Ben Abdellah University, Fez, MAR; 2 Department of Pathology, Hassan II University Hospital, Sidi Mohamed Ben Abdellah University, Fez, MAR

**Keywords:** dermoscopy, glomeruloid hemangioma, lagoons, poems syndrome, vascular tumor

## Abstract

Glomeruloid hemangioma (GH) is a rare benign vascular tumor typically associated with POEMS syndrome. Isolated GH in the absence of underlying POEMS syndrome is exceptionally uncommon and may pose a diagnostic challenge. We report the case of a 78-year-old man who presented with a slowly growing, painless, erythematous-violaceous, dome-shaped nodule measuring 3 cm on the trunk. Dermoscopy revealed lagoons separated by septa, prominent irregular linear vessels, and a blue-white veil, raising suspicion for amelanotic melanoma or other vascular tumors. Complete excisional biopsy revealed a well-circumscribed dermal vascular proliferation with a characteristic glomeruloid pattern, consisting of dilated vessels surrounded by clusters of small capillaries. Systemic evaluation, including serum protein electrophoresis, urine immunofixation, and endocrinological assessment, showed no evidence of POEMS syndrome. No recurrence was observed following surgical excision. This case highlights that GH can occur as an isolated lesion. Recognition of its dermoscopic features, particularly the *lagoons and septa* pattern, may provide valuable clues to the diagnosis and help guide appropriate management.

## Introduction

Glomeruloid hemangioma (GH) is a rare benign vascular tumor characterized by ectatic dermal capillaries with intraluminal capillary loops forming a glomerular pattern [1]. It is a classic cutaneous finding in POEMS syndrome, a multisystem disorder involving polyneuropathy, organomegaly, endocrinopathy, monoclonal gammopathy, and skin changes (hypertrichosis, sclerodermoid changes, leukonychia) [2]. However, isolated GH without POEMS syndrome is exceptionally rare [3,4] and poses a diagnostic challenge. We report a case of solitary GH unrelated to POEMS syndrome, highlighting its dermoscopic features to aid recognition of this uncommon localized vascular lesion and add to the limited data on nonsyndromic forms.

## Case presentation

A 78-year-old man with no significant medical history presented with a painless, solitary tumor on the trunk that had slowly increased in size over five months. Clinical examination revealed a firm, compressible, erythematous-violaceous, dome-shaped nodule measuring 3 cm, freely mobile over the underlying tissues (Figure 1), with no palpable regional lymphadenopathy. Dermoscopy revealed lagoons separated by septa, prominent irregular linear vessels, and a blue-white veil (Figure 2).

**Figure 1:**
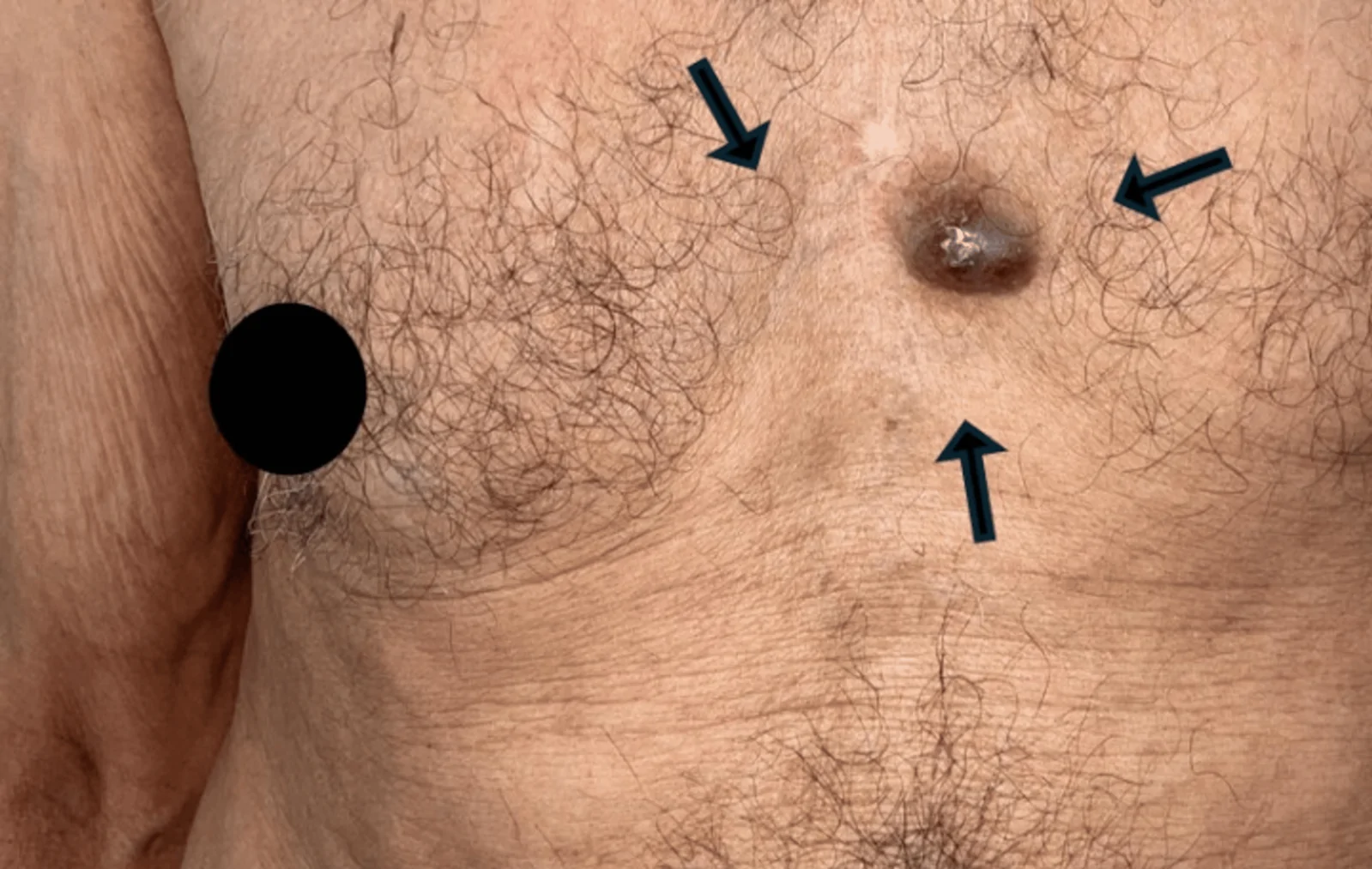
Erythematous-violaceous, dome-shaped nodule measuring 3 cm on the trunk (black arrows).

**Figure 2:**
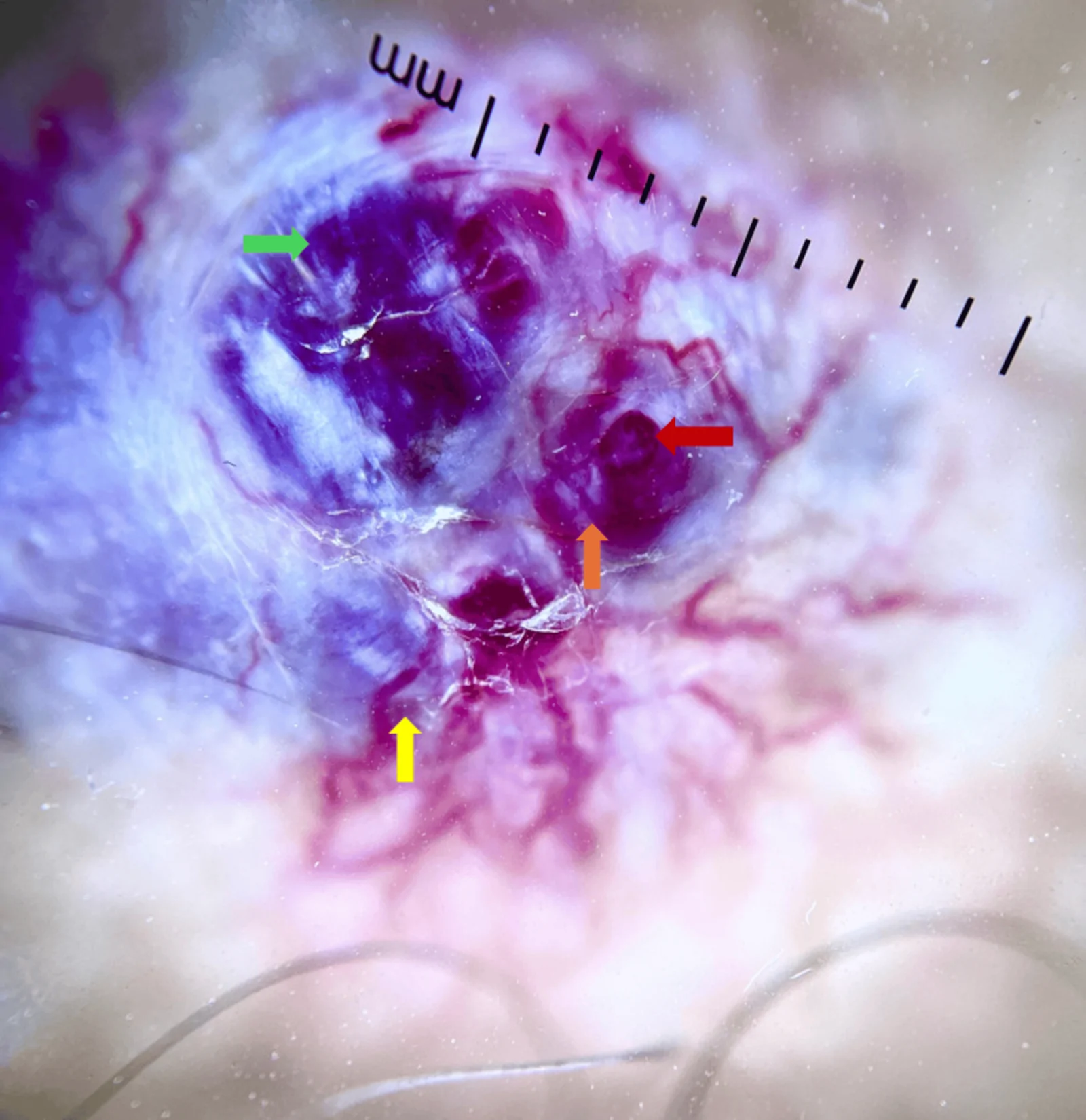
Dermoscopic features: lagoons (red arrow) separated by septa (orange arrow), irregular linear vessels (yellow arrow), and a blue-white veil (green arrow).

These findings raised suspicion for amelanotic melanoma and other vascular tumors. A complete excisional biopsy was performed. Thoracoabdominopelvic computed tomography (CT) showed no abnormalities. Routine laboratory tests, including a complete blood count and liver and renal function tests, were unremarkable. Histopathological examination revealed a well-circumscribed dermal vascular proliferation with fibrosis. The vessels varied in size, with some showing marked dilation and a glomeruloid pattern. They were lined by regular to plump endothelial cells and contained erythrocytes and occasional fibrin thrombi (Figure 3). These findings were consistent with GH.

**Figure 3:**
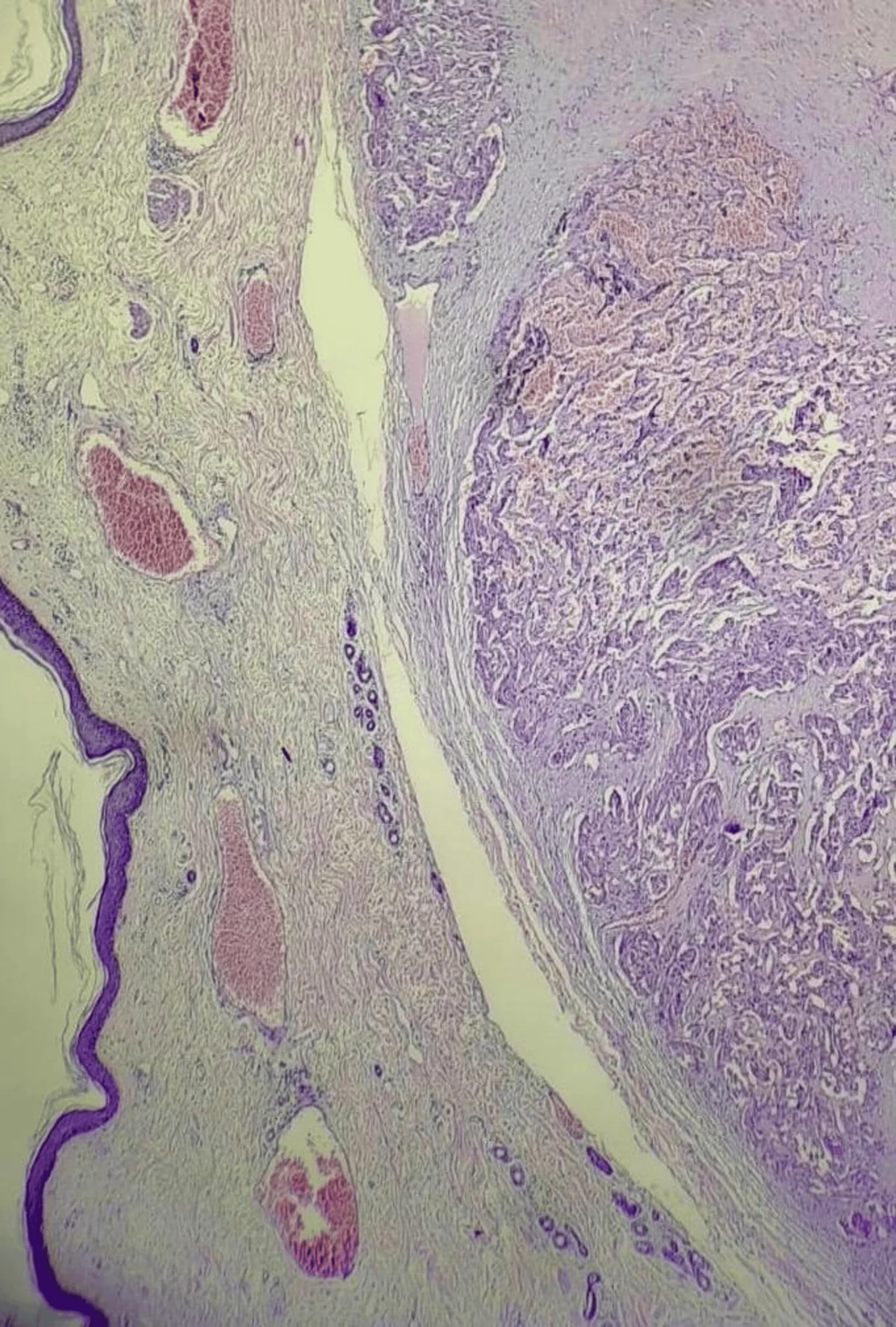
Histopathological findings. Well-circumscribed vascular proliferation of varying sizes, with some vessels showing dilation and a glomeruloid appearance. The lumina contain red blood cells and occasional fibrin thrombi (H&E stain, ×100 magnification).

At follow-up, the patient remained asymptomatic, and neurological examination was normal. In view of the potential association between GH and POEMS syndrome, an evaluation for an underlying systemic disorder was performed. Serum protein electrophoresis, urine immunofixation, and a comprehensive endocrinological evaluation were all within normal limits, with no clinical or laboratory evidence suggestive of POEMS syndrome.

## Discussion

GH was first described by Chan et al. in 1990 [3]. The precise mechanisms underlying its development remain unclear. However, the most supported hypothesis involves excessive production of proangiogenic cytokines and growth factors, including vascular endothelial growth factor (VEGF), interleukin-6, interleukin-1β, and tumor necrosis factor-α [5]. Clinically, GH presents as firm or compressible, dome-shaped papules or nodules ranging from a few millimeters to several centimeters in diameter, with red, blue, or violaceous coloration [2]. Lesions most commonly occur on the trunk or proximal extremities, although the face may occasionally be involved [3]. Dermoscopy has been infrequently described in GH but may represent a useful, rapid, and non-invasive diagnostic tool. Reported dermoscopic features include irregular linear vessels, red lacunae, and red-white structureless areas [6]. In our patient, in addition to these previously described vascular features, we observed a blue-white veil, initially raising suspicion for amelanotic melanoma. The dermoscopic differential diagnosis of vascular lesions includes several entities with overlapping vascular and lacunar features, such as angiomas, angiokeratomas, pyogenic granulomas, intravascular papillary endothelial hyperplasia (Masson tumor), and pseudo-Kaposi sarcoma [7,8]. These lesions may show variable combinations of lacunae, vascular structures, and hemorrhagic or whitish areas, which can complicate their distinction from other vascular tumors and malignant mimickers [7,8]. The reported spectrum of non-Kaposi vascular lesions with overlapping dermoscopic features further supports considering these entities in the differential diagnosis [9]. In our case, the combination of the previously described vascular features of GH with an unusual blue-white veil broadened the dermoscopic differential diagnosis and ultimately prompted histopathological confirmation. This finding further illustrates the dermoscopic variability of GH and expands the spectrum of dermoscopic features reported for this uncommon vascular tumor.

Histologically, GH is characterized by a well-circumscribed, lobulated vascular proliferation within the dermis. A central dilated vessel with a sinusoidal or slit-like appearance is surrounded by clusters of small capillaries arranged in a characteristic glomeruloid pattern. The endothelial cells are flat to plump but cytologically bland, lining vascular lumina containing erythrocytes and occasional fibrin thrombi. The surrounding stroma may show fibrosis, contributing to the lesion's characteristic architecture [2].

GH is reported in approximately 26%-44% of patients with POEMS syndrome [3]. Although strongly associated with POEMS syndrome, GH may rarely occur as an isolated cutaneous lesion in patients without evidence of an underlying systemic disorder [5]. Our case represents such an uncommon non-syndromic presentation. Given the recognized association between GH and POEMS syndrome, the absence of neurological abnormalities and the normal results of serum protein electrophoresis, urine immunofixation, and endocrinological evaluation in our patient are important findings.

Treatment of GH depends on the clinical context and the presence or absence of an underlying systemic disorder. Reported therapeutic approaches include surgical excision, intralesional triamcinolone, systemic corticosteroids, thalidomide, and laser therapy [3]. In our patient, complete surgical excision was performed, with no recurrence or symptoms reported during follow-up.

## Conclusions

This case highlights that GH can occur as an isolated lesion in the absence of POEMS syndrome. Although uncommon, this entity should be considered in the differential diagnosis of solitary vascular tumors, particularly when located on the trunk. Dermoscopic features, especially the presence of a lagoons and septa pattern associated with prominent vascular structures, may provide valuable clues to the diagnosis. Nevertheless, histopathological examination remains essential for diagnostic confirmation, and an appropriate systemic evaluation should always be performed to investigate the possibility of an underlying disorder.
